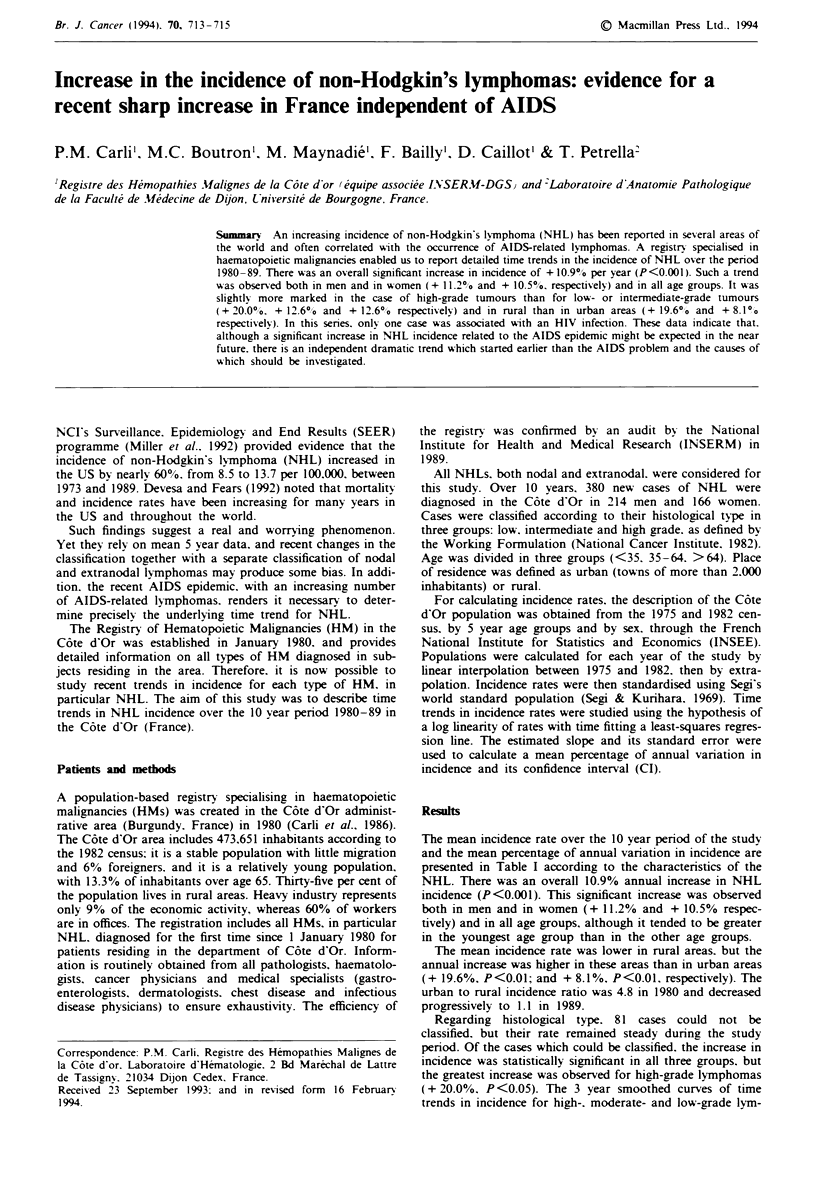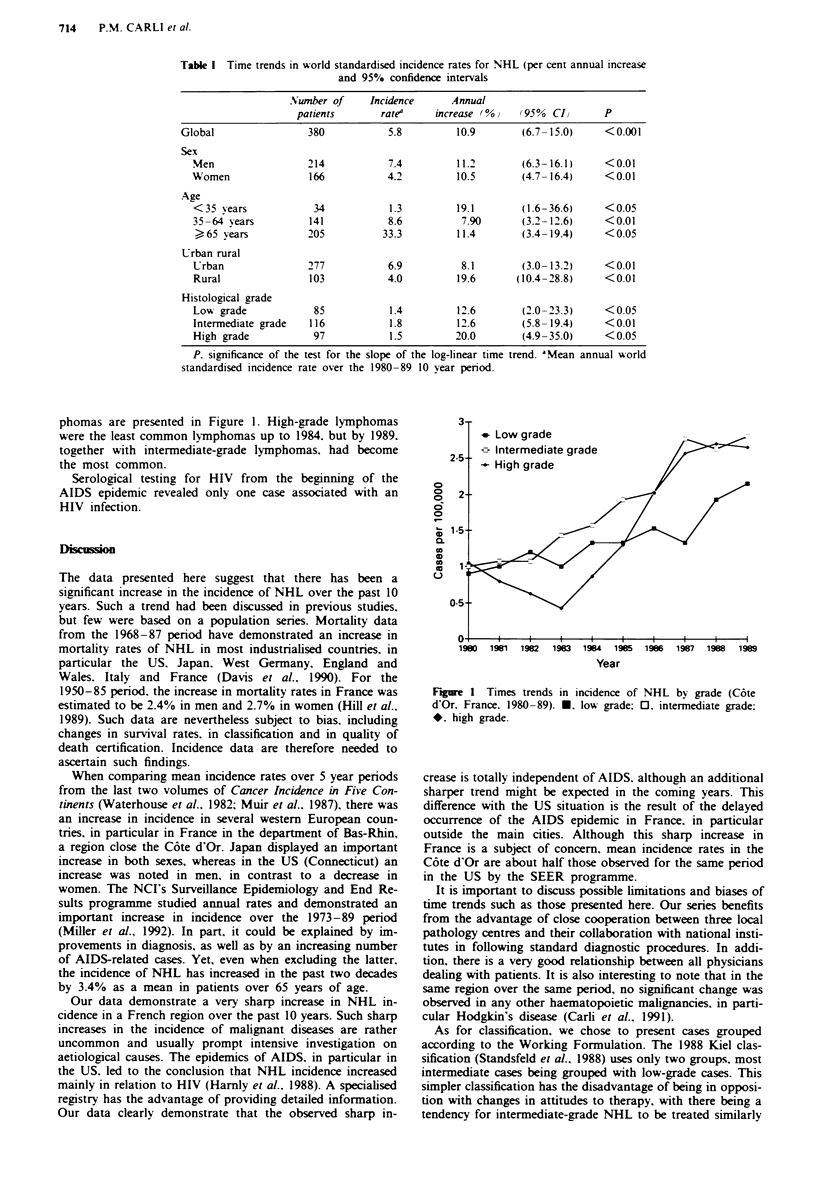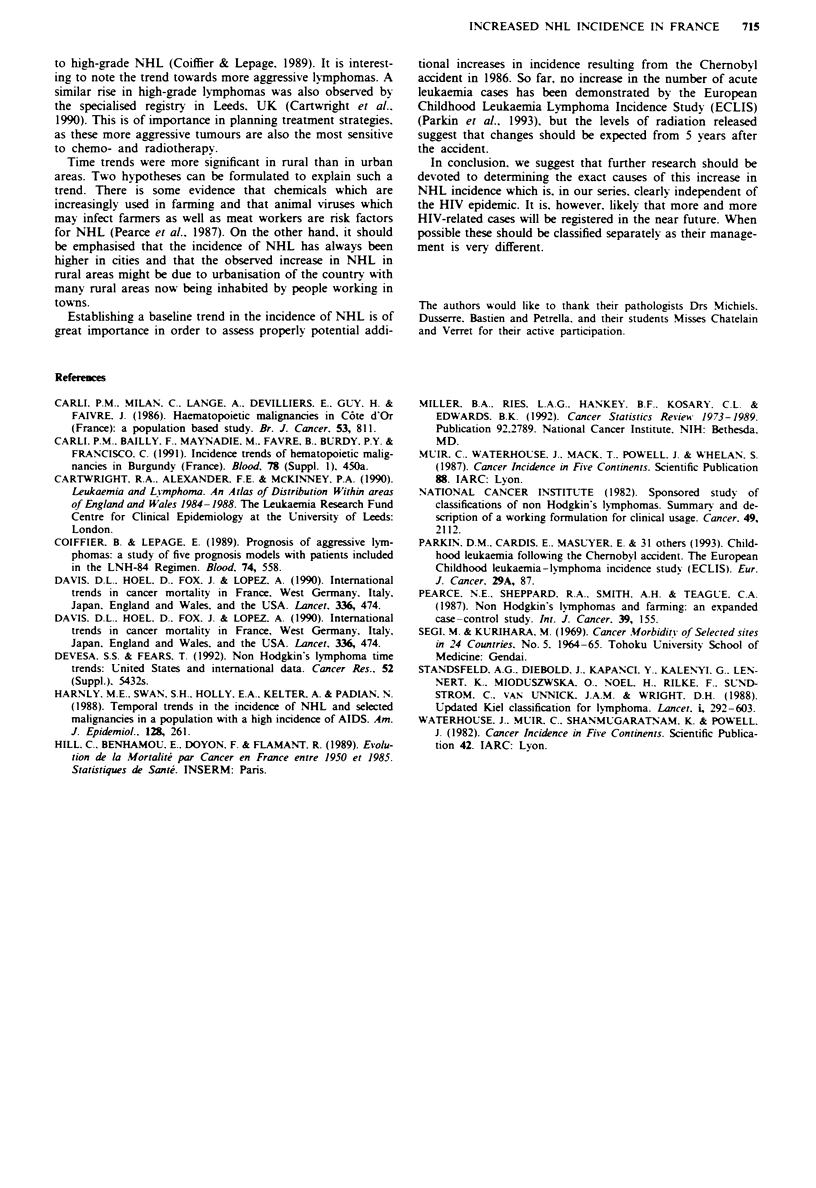# Increase in the incidence of non-Hodgkin's lymphomas: evidence for a recent sharp increase in France independent of AIDS.

**DOI:** 10.1038/bjc.1994.380

**Published:** 1994-10

**Authors:** P. M. Carli, M. C. Boutron, M. Maynadié, F. Bailly, D. Caillot, T. Petrella

**Affiliations:** Registre des Hémopathies Malignes de la Côte d'or (équipe associée INSERM-DGS), France.

## Abstract

An increasing incidence of non-Hodgkin's lymphoma (NHL) has been reported in several areas of the world and often correlated with the occurrence of AIDS-related lymphomas. A registry specialised in haematopoietic malignancies enabled us to report detailed time trends in the incidence of NHL over the period 1980-89. There was an overall significant increase in incidence of + 10.9% per year (P < 0.001). Such a trend was observed both in men and in women (+ 11.2% and + 10.5%, respectively) and in all age groups. It was slightly more marked in the case of high-grade tumours than for low- or intermediate-grade tumours (+ 20.0%, + 12.6% and + 12.6% respectively) and in rural than in urban areas (+ 19.6% and + 8.1% respectively). In this series, only one case was associated with an HIV infection. These data indicate that, although a significant increase in NHL incidence related to the AIDS epidemic might be expected in the near future, there is an independent dramatic trend which started earlier than the AIDS problem and the causes of which should be investigated.


Br. J. Cancer (1994), 70, 713-715 © Macmillan Press Ltd., 1994

### Increase in the incidence of non-Hodgkin's lymphomas: evidence for a recent sharp increase in France independent of AIDS

P.M. Carli[1], M.C. Boutron[1], M. Maynadié[1], F. Bailly[1], D. Caillot[1] & T. Petrella[2]

[1]*Registre des Hémopathies Malignes de la Côte d'or (équipe associée INSERM-DGS) and* [2]*Laboratoire d'Anatomie Pathologique de la Faculté de Médecine de Dijon, Université de Bourgogne, France.*

**Summary** An increasing incidence of non-Hodgkin's lymphoma (NHL) has been reported in several areas of the world and often correlated with the occurrence of AIDS-related lymphomas. A registry specialised in haematopoietic malignancies enabled us to report detailed time trends in the incidence of NHL over the period 1980-89. There was an overall significant increase in incidence of +10.9% per year ($P<0.001$). Such a trend was observed both in men and in women (+11.2% and +10.5%, respectively) and in all age groups. It was slightly more marked in the case of high-grade tumours than for low- or intermediate-grade tumours (+20.0%, +12.6% and +12.6% respectively) and in rural than in urban areas (+19.6% and +8.1% respectively). In this series, only one case was associated with an HIV infection. These data indicate that, although a significant increase in NHL incidence related to the AIDS epidemic might be expected in the near future, there is an independent dramatic trend which started earlier than the AIDS problem and the causes of which should be investigated.

NCI's Surveillance, Epidemiology and End Results (SEER) programme (Miller *et al.*, 1992) provided evidence that the incidence of non-Hodgkin's lymphoma (NHL) increased in the US by nearly 60%, from 8.5 to 13.7 per 100,000, between 1973 and 1989. Devesa and Fears (1992) noted that mortality and incidence rates have been increasing for many years in the US and throughout the world.

Such findings suggest a real and worrying phenomenon. Yet they rely on mean 5 year data, and recent changes in the classification together with a separate classification of nodal and extranodal lymphomas may produce some bias. In addition, the recent AIDS epidemic, with an increasing number of AIDS-related lymphomas, renders it necessary to determine precisely the underlying time trend for NHL.

The Registry of Hematopoietic Malignancies (HM) in the Côte d'Or was established in January 1980, and provides detailed information on all types of HM diagnosed in subjects residing in the area. Therefore, it is now possible to study recent trends in incidence for each type of HM, in particular NHL. The aim of this study was to describe time trends in NHL incidence over the 10 year period 1980-89 in the Côte d'Or (France).

### Patients and methods

A population-based registry specialising in haematopoietic malignancies (HMs) was created in the Côte d'Or administrative area (Burgundy, France) in 1980 (Carli *et al.*, 1986). The Côte d'Or area includes 473,651 inhabitants according to the 1982 census; it is a stable population with little migration and 6% foreigners, and it is a relatively young population, with 13.3% of inhabitants over age 65. Thirty-five per cent of the population lives in rural areas. Heavy industry represents only 9% of the economic activity, whereas 60% of workers are in offices. The registration includes all HMs, in particular NHL, diagnosed for the first time since 1 January 1980 for patients residing in the department of Côte d'Or. Information is routinely obtained from all pathologists, haematologists, cancer physicians and medical specialists (gastroenterologists, dermatologists, chest disease and infectious disease physicians) to ensure exhaustivity. The efficiency of the registry was confirmed by an audit by the National Institute for Health and Medical Research (INSERM) in 1989.

All NHLs, both nodal and extranodal, were considered for this study. Over 10 years, 380 new cases of NHL were diagnosed in the Côte d'Or in 214 men and 166 women. Cases were classified according to their histological type in three groups: low, intermediate and high grade, as defined by the Working Formulation (National Cancer Institute, 1982). Age was divided in three groups (<35, 35-64, >64). Place of residence was defined as urban (towns of more than 2,000 inhabitants) or rural.

For calculating incidence rates, the description of the Côte d'Or population was obtained from the 1975 and 1982 census, by 5 year age groups and by sex, through the French National Institute for Statistics and Economics (INSEE). Populations were calculated for each year of the study by linear interpolation between 1975 and 1982, then by extrapolation. Incidence rates were then standardised using Segi's world standard population (Segi & Kurihara, 1969). Time trends in incidence rates were studied using the hypothesis of a log linearity of rates with time fitting a least-squares regression line. The estimated slope and its standard error were used to calculate a mean percentage of annual variation in incidence and its confidence interval (CI).

### Results

The mean incidence rate over the 10 year period of the study and the mean percentage of annual variation in incidence are presented in Table I according to the characteristics of the NHL. There was an overall 10.9% annual increase in NHL incidence ($P<0.001$). This significant increase was observed both in men and in women (+11.2% and +10.5% respectively) and in all age groups, although it tended to be greater in the youngest age group than in the other age groups.

The mean incidence rate was lower in rural areas, but the annual increase was higher in these areas than in urban areas (+19.6%, $P<0.01$; and +8.1%, $P<0.01$, respectively). The urban to rural incidence ratio was 4.8 in 1980 and decreased progressively to 1.1 in 1989.

Regarding histological type, 81 cases could not be classified, but their rate remained steady during the study period. Of the cases which could be classified, the increase in incidence was statistically significant in all three groups, but the greatest increase was observed for high-grade lymphomas (+20.0%, $P<0.05$). The 3 year smoothed curves of time trends in incidence for high-, moderate- and low-grade lym-

Correspondence: P.M. Carli, Registre des Hémopathies Malignes de la Côte d'or, Laboratoire d'Hématologie, 2 Bd Maréchal de Lattre de Tassigny, 21034 Dijon Cedex, France.
Received 23 September 1993; and in revised form 16 February 1994.

**Table I** Time trends in world standardised incidence rates for NHL (per cent annual increase and 95% confidence intervals

| | *Number of patients* | *Incidence rate*[a] | *Annual increase (%)* | *(95% CI)* | *P* |
|---|---|---|---|---|---|
| Global | 380 | 5.8 | 10.9 | (6.7-15.0) | <0.001 |
| Sex | | | | | |
| Men | 214 | 7.4 | 11.2 | (6.3-16.1) | <0.01 |
| Women | 166 | 4.2 | 10.5 | (4.7-16.4) | <0.01 |
| Age | | | | | |
| <35 years | 34 | 1.3 | 19.1 | (1.6-36.6) | <0.05 |
| 35-64 years | 141 | 8.6 | 7.90 | (3.2-12.6) | <0.01 |
| ≥65 years | 205 | 33.3 | 11.4 | (3.4-19.4) | <0.05 |
| Urban rural | | | | | |
| Urban | 277 | 6.9 | 8.1 | (3.0-13.2) | <0.01 |
| Rural | 103 | 4.0 | 19.6 | (10.4-28.8) | <0.01 |
| Histological grade | | | | | |
| Low grade | 85 | 1.4 | 12.6 | (2.0-23.3) | <0.05 |
| Intermediate grade | 116 | 1.8 | 12.6 | (5.8-19.4) | <0.01 |
| High grade | 97 | 1.5 | 20.0 | (4.9-35.0) | <0.05 |

*P*, significance of the test for the slope of the log-linear time trend. [a]Mean annual world standardised incidence rate over the 1980-89 10 year period.

phomas are presented in Figure 1. High-grade lymphomas were the least common lymphomas up to 1984, but by 1989, together with intermediate-grade lymphomas, had become the most common.

Serological testing for HIV from the beginning of the AIDS epidemic revealed only one case associated with an HIV infection.

### Discussion

The data presented here suggest that there has been a significant increase in the incidence of NHL over the past 10 years. Such a trend had been discussed in previous studies, but few were based on a population series. Mortality data from the 1968-87 period have demonstrated an increase in mortality rates of NHL in most industrialised countries, in particular the US, Japan, West Germany, England and Wales, Italy and France (Davis *et al.*, 1990). For the 1950-85 period, the increase in mortality rates in France was estimated to be 2.4% in men and 2.7% in women (Hill *et al.*, 1989). Such data are nevertheless subject to bias, including changes in survival rates, in classification and in quality of death certification. Incidence data are therefore needed to ascertain such findings.

When comparing mean incidence rates over 5 year periods from the last two volumes of *Cancer Incidence in Five Continents* (Waterhouse *et al.*, 1982; Muir *et al.*, 1987), there was an increase in incidence in several western European countries, in particular in France in the department of Bas-Rhin, a region close the Côte d'Or. Japan displayed an important increase in both sexes, whereas in the US (Connecticut) an increase was noted in men, in contrast to a decrease in women. The NCI's Surveillance Epidemiology and End Results programme studied annual rates and demonstrated an important increase in incidence over the 1973-89 period (Miller *et al.*, 1992). In part, it could be explained by improvements in diagnosis, as well as by an increasing number of AIDS-related cases. Yet, even when excluding the latter, the incidence of NHL has increased in the past two decades by 3.4% as a mean in patients over 65 years of age.

Our data demonstrate a very sharp increase in NHL incidence in a French region over the past 10 years. Such sharp increases in the incidence of malignant diseases are rather uncommon and usually prompt intensive investigation on aetiological causes. The epidemics of AIDS, in particular in the US, led to the conclusion that NHL incidence increased mainly in relation to HIV (Harnly *et al.*, 1988). A specialised registry has the advantage of providing detailed information. Our data clearly demonstrate that the observed sharp in-

**Figure 1** Times trends in incidence of NHL by grade (Côte d'Or, France, 1980-89). ■, low grade; □, intermediate grade; ◆, high grade.

crease is totally independent of AIDS, although an additional sharper trend might be expected in the coming years. This difference with the US situation is the result of the delayed occurrence of the AIDS epidemic in France, in particular outside the main cities. Although this sharp increase in France is a subject of concern, mean incidence rates in the Côte d'Or are about half those observed for the same period in the US by the SEER programme.

It is important to discuss possible limitations and biases of time trends such as those presented here. Our series benefits from the advantage of close cooperation between three local pathology centres and their collaboration with national institutes in following standard diagnostic procedures. In addition, there is a very good relationship between all physicians dealing with patients. It is also interesting to note that in the same region over the same period, no significant change was observed in any other haematopoietic malignancies, in particular Hodgkin's disease (Carli *et al.*, 1991).

As for classification, we chose to present cases grouped according to the Working Formulation. The 1988 Kiel classification (Standsfeld *et al.*, 1988) uses only two groups, most intermediate cases being grouped with low-grade cases. This simpler classification has the disadvantage of being in opposition with changes in attitudes to therapy, with there being a tendency for intermediate-grade NHL to be treated similarly to high-grade NHL (Coiffier & Lepage, 1989). It is interesting to note the trend towards more aggressive lymphomas. A similar rise in high-grade lymphomas was also observed by the specialised registry in Leeds, UK (Cartwright *et al.*, 1990). This is of importance in planning treatment strategies, as these more aggressive tumours are also the most sensitive to chemo- and radiotherapy.

Time trends were more significant in rural than in urban areas. Two hypotheses can be formulated to explain such a trend. There is some evidence that chemicals which are increasingly used in farming and that animal viruses which may infect farmers as well as meat workers are risk factors for NHL (Pearce *et al.*, 1987). On the other hand, it should be emphasised that the incidence of NHL has always been higher in cities and that the observed increase in NHL in rural areas might be due to urbanisation of the country with many rural areas now being inhabited by people working in towns.

Establishing a baseline trend in the incidence of NHL is of great importance in order to assess properly potential additional increases in incidence resulting from the Chernobyl accident in 1986. So far, no increase in the number of acute leukaemia cases has been demonstrated by the European Childhood Leukaemia Lymphoma Incidence Study (ECLIS) (Parkin *et al.*, 1993), but the levels of radiation released suggest that changes should be expected from 5 years after the accident.

In conclusion, we suggest that further research should be devoted to determining the exact causes of this increase in NHL incidence which is, in our series, clearly independent of the HIV epidemic. It is, however, likely that more and more HIV-related cases will be registered in the near future. When possible these should be classified separately as their management is very different.

The authors would like to thank their pathologists Drs Michiels, Dusserre, Bastien and Petrella, and their students Misses Chatelain and Verret for their active participation.

**References**

CARLI, P.M., MILAN, C., LANGE, A., DEVILLIERS, E., GUY, H. & FAIVRE, J. (1986). Haematopoietic malignancies in Côte d'Or (France): a population based study. *Br. J. Cancer*, **53**, 811.

CARLI, P.M., BAILLY, F., MAYNADIE, M., FAVRE, B., BURDY, P.Y. & FRANCISCO, C. (1991). Incidence trends of hematopoietic malignancies in Burgundy (France). *Blood*, **78** (Suppl. 1), 450a.

CARTWRIGHT, R.A., ALEXANDER, F.E. & MCKINNEY, P.A. (1990). *Leukaemia and Lymphoma. An Atlas of Distribution Within areas of England and Wales 1984–1988*. The Leukaemia Research Fund Centre for Clinical Epidemiology at the University of Leeds: London.

COIFFIER, B. & LEPAGE, E. (1989). Prognosis of aggressive lymphomas: a study of five prognosis models with patients included in the LNH-84 Regimen. *Blood*, **74**, 558.

DAVIS, D.L., HOEL, D., FOX, J. & LOPEZ, A. (1990). International trends in cancer mortality in France, West Germany, Italy, Japan, England and Wales, and the USA. *Lancet*, **336**, 474.

DAVIS, D.L., HOEL, D., FOX, J. & LOPEZ, A. (1990). International trends in cancer mortality in France, West Germany, Italy, Japan, England and Wales, and the USA. *Lancet*, **336**, 474.

DEVESA, S.S. & FEARS, T. (1992). Non Hodgkin's lymphoma time trends: United States and international data. *Cancer Res.*, **52** (Suppl.), 5432s.

HARNLY, M.E., SWAN, S.H., HOLLY, E.A., KELTER, A. & PADIAN, N. (1988). Temporal trends in the incidence of NHL and selected malignancies in a population with a high incidence of AIDS. *Am. J. Epidemiol.*, **128**, 261.

HILL, C., BENHAMOU, E., DOYON, F. & FLAMANT, R. (1989). *Evolution de la Mortalité par Cancer en France entre 1950 et 1985. Statistiques de Santé*. INSERM: Paris.

MILLER, B.A., RIES, L.A.G., HANKEY, B.F., KOSARY, C.L. & EDWARDS, B.K. (1992). *Cancer Statistics Review 1973–1989*. Publication 92.2789. National Cancer Institute, NIH: Bethesda, MD.

MUIR, C., WATERHOUSE, J., MACK, T., POWELL, J. & WHELAN, S. (1987). *Cancer Incidence in Five Continents*. Scientific Publication **88**. IARC: Lyon.

NATIONAL CANCER INSTITUTE (1982). Sponsored study of classifications of non Hodgkin's lymphomas. Summary and description of a working formulation for clinical usage. *Cancer*, **49**, 2112.

PARKIN, D.M., CARDIS, E., MASUYER, E. & 31 others (1993). Childhood leukaemia following the Chernobyl accident. The European Childhood leukaemia-lymphoma incidence study (ECLIS). *Eur. J. Cancer*, **29A**, 87.

PEARCE, N.E., SHEPPARD, R.A., SMITH, A.H. & TEAGUE, C.A. (1987). Non Hodgkin's lymphomas and farming: an expanded case-control study. *Int. J. Cancer*, **39**, 155.

SEGI, M. & KURIHARA, M. (1969). *Cancer Morbidity of Selected sites in 24 Countries*, No. 5, 1964–65. Tohoku University School of Medicine: Gendai.

STANDSFELD, A.G., DIEBOLD, J., KAPANCI, Y., KALENYI, G., LENNERT, K., MIODUSZWSKA, O., NOEL, H., RILKE, F., SUNDSTROM, C., VAN UNNICK, J.A.M. & WRIGHT, D.H. (1988). Updated Kiel classification for lymphoma. *Lancet*, **i**, 292–603.

WATERHOUSE, J., MUIR, C., SHANMUGARATNAM, K. & POWELL, J. (1982). *Cancer Incidence in Five Continents*. Scientific Publication **42**. IARC: Lyon.